# Atypical long-latency auditory event-related potentials in a subset of children with specific language impairment

**DOI:** 10.1111/j.1467-7687.2007.00620.x

**Published:** 2007-09

**Authors:** Dorothy VM Bishop, Mervyn Hardiman, Ruth Uwer, Waldemar von Suchodoletz

**Affiliations:** 1 Department of Experimental Psychology, University of Oxford UK; 2 Department of Child and Adolescent Psychiatry and Psychotherapy, University of Munich Germany

## Abstract

It has been proposed that specific language impairment (SLI) is the consequence of low-level abnormalities in auditory perception. However, studies of long-latency auditory ERPs in children with SLI have generated inconsistent findings. A possible reason for this inconsistency is the heterogeneity of SLI. The intraclass correlation (ICC) has been proposed as a useful statistic for evaluating heterogeneity because it allows one to compare an individual's auditory ERP with the grand average waveform from a typically developing reference group. We used this method to reanalyse auditory ERPs from a sample previously described by Uwer, Albrecht and von Suchodoletz (2002). In a subset of children with receptive SLI, there was less correspondence (i.e. lower ICC) with the normative waveform (based on the control grand average) than for typically developing children. This poorer correspondence was seen in responses to both tone and speech stimuli for the period 100–228 ms post stimulus onset. The effect was lateralized and seen at right- but not left-sided electrodes.

## Introduction

Specific language impairment (SLI), also known as developmental language disorder, is diagnosed when a child has difficulty in producing or understanding spoken language for no apparent reason. According to ICD-10 criteria (World Health Organization, 1993), the diagnosis is made when language development is out of keeping with other aspects of development, and possible explanatory causes have been excluded. Prevalence figures vary depending on how stringently such criteria are applied, and whether the focus is just on receptive language impairment, or is extended to include those with primarily expressive language difficulties. In an extensive epidemiological study in the USA, Tomblin, Records, Buckwalter, Zhang, Smith and O’Brien (1997) estimated the prevalence in 5- to 6-year-olds at around 7%, although the percentage of children whose problems attract clinical concern is lower than this.

Over the past few decades, considerable progress has been made in understanding the etiology of SLI, and it is now widely recognized that the causes are predominantly neurobiological rather than sociocultural, with genes playing a substantial role (see Bishop, 2002, for review). Nevertheless, we have made remarkably little progress in understanding the neurological basis of SLI. In part this may be because the number of studies using direct measures of neurological structure or function is small, but another reason is the heterogeneity of the disorder. Traditionally, SLI has been subdivided into expressive vs. expressive-receptive subtypes (American Psychiatric Association, 1994), though this gross contrast may be inadequate for capturing the full range of clinical variation (Bishop, 2004). It seems likely that different etiologies are implicated in different subtypes of SLI. Certainly, there is wide variation in results from neuroimaging studies. For instance, in studies using structural MRI, Jernigan, Hesselink, Sowell and Tallal (1991) and Leonard, Lombardino, Walsh, Eckert, Mockler, Rowe, Williams and DeBose (2002) reported that SLI was associated with small cerebral volume, but Herbert *et al.* (Herbert, Ziegler, Makris, Bakardjiev, Hodgson, Adrien, Kennedy, Filipek & Caviness, 2003) found just the opposite. Abnormalities of gyral development in frontal and temporal cortical regions are rather more common in people with SLI than in unaffected individuals, but the relationship is weak and probabilistic (Plante & Jackson, 1997; Clark & Plante, 1998). Such findings suggest that we need to use methods that allow us to identify neurobiological abnormalities at the level of the individual, rather than averaging results across a group. One line of evidence comes from electrophysiological studies of auditory event-related potentials (ERPs). With ERPs we can obtain an index of the brain's responses to auditory stimuli in real time while minimizing performance demands on the child.

To date, there have been a handful of studies looking at passively elicited auditory ERPs in children with SLI vs. controls, but analysis has largely focused on comparing mean amplitudes and latencies of ERP peaks at the group level. The P1, N1 and P2 peaks have been a particular focus of interest, because they provide a window on the earliest stages of processing in auditory cortex: if it were possible to demonstrate abnormal brain responses at this level, this would provide evidence against the view that perceptual abnormalities in SLI are confined to linguistically relevant stimuli (e.g. Nittrouer, 2002). Results, however, have been inconsistent. Mason and Mellor (1984), using a slow presentation rate (2000 ms stimulus onset asynchrony; SOA) found no abnormalities of N1 and P2 latency or amplitude at central or temporal sites in eight children with SLI, when compared with 12 controls, although there was some suggestion of atypical lateralization of responses in temporal regions in the children with SLI. The sample was not very well specified, with few details of language and cognitive test results. Other studies that found no differences in amplitude or latency of N1 and P2 when comparing children with SLI and controls include Lincoln, Courchesne, Harms and Allen (1995) (electrode Cz with SOA of 2000 ms) and Marler, Champlin and Gillam (2002) (electrodes C3, Cz and C4, with SOA of 500 or 1000 ms). These studies contrast with results from Korpilahti and Lang (1994) and Tonnquist-Uhlén and colleagues (Tonnquist-Uhlén, 1996a, 1996b; Tonnquist-Uhlén, Borg, Persson & Spens, 1996). Korpilahti and Lang (1994) were not able to compare N1 and P2 in their sample of 14 children with SLI and 12 controls because they used a rapid presentation rate (SOA 450 ms) and these peaks were often not present, but they did report a significant delay in the latency of N2 in those with SLI. However, the control group was apparently not given any psychological tests, and thus was not matched to the SLI group on nonverbal ability.

Tonnquist-Uhlén and colleagues (Tonnquist-Uhlén, 1996a, 1996b; Tonnquist-Uhlén *et al.*, 1996) examined N1, P2, N2 and the T-complex, for a 500 Hz tone presented with SOA of 900 ms. They used topographic analyses as well as measures of peak amplitude and latency. Unfortunately, the psychological description of their sample is sketchy, and so it is not possible to establish whether the control group was matched, or indeed assessed for language and cognitive function. Nor can one tell how severe the language disorders were, nor whether all children scored within normal limits on performance IQ; it is more appropriate therefore to refer to these as children with language impairment (LI) rather than SLI. Nine of the 20 participants with LI were judged to have pathological EEGs (Bø, Marklund, Hamsten, Persson & Tonnquist-Uhlén, 1992). Tonnquist-Uhlén *et al.* (1996; Tonnquist-Uhlén, 1996a) noted that the overall topography of N1 and P2 was generally similar in LI and control groups, but both peaks were significantly later in those with LI compared to 20 controls, this effect being seen for N1 only after right-ear stimulation. In addition, topographic maps were compared to a control grand average for the time window corresponding to N1, and a higher rate of deviant topography was seen in the LI group. Overall, it was concluded that when information on N1 latency, amplitude and topography were combined, the majority of those with abnormal results were in the LI group. Nevertheless, many of the LI children had waveforms comparable to those seen in controls. An intriguing feature of these data was that the latency of N1 decreased with age (from 9 to 15 years) in the control group, but not in the LI group, and inspection of the scatterplots indicates that this is because the N1 latencies of the older LI children (aged 13 to 16 years) were more similar to those of younger control children than to their own age group. The authors suggested that this could indicate a persistent abnormality or a delay in maturation. Delayed maturation was also suggested as an explanation of findings by Jirsa and Clontz (1990), who studied a group of children who had language impairments accompanied by poor scores on a central auditory processing test battery. Unlike the other studies reviewed here, they adopted an active paradigm, with ERPs recorded as children pressed a button when detecting a deviant tone. They found that 12/18 children in the clinical group had N1 latencies more than 2 SE from a control mean, and 8/18 had equivalent delays in P2. No controls had abnormal N1 latency and only one had abnormal P2. However, in a similar active paradigm, Ors, Lindgren, Blennow, Nettelbladt, Sahlén and Rosén (2002) failed to find any group differences in N1-P2 in a comparison of 10 children with SLI and 10 control children aged from 10 to 14 years.

Neville, Coffey, Holcomb and Tallal (1993) suggested that inconsistent findings might reflect heterogeneity in the SLI population, with only a subset of children having auditory processing problems. In a complex experiment where tones were presented at different SOAs, they found no overall difference between 22 children with SLI and 12 control children for amplitude or latency of N1 and P2. However, when children were subdivided according to their performance on an auditory temporal processing task, those with low scores showed a trend for delayed N1, especially over the parietal and temporal regions of the left hemisphere. This supports the idea that we need to move away from group analyses and develop methods for identifying abnormality of ERPs in individual children.

The goal of analysing auditory ERPs in individual children is complicated by the fact that at fast presentation rates, N1 and P2 peaks are often not seen in ERPs of children under 12 years of age (Albrecht, von Suchodoletz & Uwer, 2000; Ponton, Eggermont, Kwong & Don, 2000). Thus if we want to study early cortical responses to sounds in children we need a method that does not depend on identifying specific peaks.

McArthur and Bishop's (2004) solution to the latter problem was to use a method for analysing auditory ERPs that enabled one to establish how far a child's waveform was age-appropriate, without requiring that specific peaks be identified. They used the intraclass correlation (ICC) statistic to quantify the extent of similarity between an individual's auditory ERP and a normative grand average. The ICC gives a global index of similarity between two waveforms and is sensitive to both amplitude and latency differences in peaks and troughs. In an initial study using this method, McArthur and Bishop (2004) showed that young people with SLI had lower ICCs than a group of matched controls. In a follow-up study using many of the same participants, Bishop and McArthur (2005) replicated this finding. Overall, these studies suggested that the ICC measure might be more sensitive than conventional measures of peak latency and amplitude in identifying cases where the auditory ERP was not age-appropriate. However, the study by McArthur and Bishop (2004) included only 16 participants with SLI aged from 10 to 19 years and 16 controls matched for age and IQ. The authors noted that the generalizability of their findings needed to be tested against a much more substantial normative background. A sample of children with SLI (Uwer *et al.*, 2002) provided an opportunity to do this. We focused on the time period 100–228 ms post stimulus onset, as this was the interval over which McArthur and Bishop (2004) had found atypical ERPs in their SLI group.

## Methods

### Participants

The data came from Uwer *et al.* (2002) and Albrecht *et al.* (2000). The characteristics of typically developing and SLI samples is described in detail by Uwer *et al.* (2002) and summarized in Table 1. In brief, children with SLI were aged from 5 to 10 years and either attended special schools for children with language impairments, or had been referred to a clinic for developmental problems. All had nonverbal IQs of 85 or above (receptive SLI mean = 98, SD = 10.1; expressive SLI mean = 103, SD = 11.2). Children with receptive SLI scored 2 SD or more below the normative mean on a test of grammatical comprehension, and those with expressive SLI scored this poorly on a test of imitating grammatical structures. The typically developing sample consisted of 5- to 10-year-old children matched in age to the SLI sample and with nonverbal IQ in the normal range (mean 108, SD = 11.2). Individuals with psychiatric disorders, peripheral hearing loss or neurological impairment were excluded from the sample. All the control children in this sample were included in the larger sample of typically developing children analysed by Bishop *et al.* (see Bishop, Hardiman, Uwer & von Suchodoletz, 2007).

**Table 1:** Numbers of females and males in typically developing (TD) and SLI groups, by age

	TD			SLI		
		
	Female	Male	Total	Female	Male	Total
5 to 6 years	3	8	11	3	5	8
7 to 8 years	8	12	20	3	20	23
9 to 10 years	9	7	16	3	8	11
Total	20	27	47	9	33	42

### Behavioural test of auditory discrimination

As described more fully by Uwer *et al.* (2002), all children with SLI and 16 of their matched controls were tested for their ability to discriminate the standard and deviant stimuli used in the ERP session. They were played pairs of stimuli and asked to judge if they were the ‘same’ or ‘different’. For each of four contrasts (tones contrasting in frequency or duration, and /da/ vs. /ba/ or /ga/) 15 same and 15 different pairs were presented. The total number of errors was recorded.

### Electrophysiological recording procedure

ERPs were recorded as participants were passively presented with either tones or speech syllables played in four blocks each of 333 stimuli with constant stimulus-onset asynchrony of 1 second. An oddball paradigm was used in which a standard stimulus was presented on 70% of trials and one of two deviants on the remaining trials; however, the analyses reported here are concerned solely with responses to standard stimuli. The standard tone stimulus was a 1000 Hz sinusoid (rise and fall time 10 ms) and the speech stimulus was a digitized syllable /da/ spoken by a female German speaker. Both were of 175 ms duration. Stimuli were presented to the right ear via earphones at 86 dB SPL. During the recording, children watched a silent videotape and were instructed to ignore the tones.

Data were acquired using a Neuroscan system with sampling rate of 256 Hz, with recordings from silver silver-chloride electrodes positioned according to the International 10/20 system at 22 sites: FP1, FP2, F3, F4, F7, F8, FZ, FT9, FT10, T3, T4, T5, T6, C3, C4, CZ, P3, P4, PZ, O1, O2, OZ. (Note that in the more contemporary 10/10 system, T3, T4, T5 and T6 are referred to as T7, T8, P7 and P8, respectively.) Electrodes were referenced to the right mastoid, and horizontal (HEOG) and vertical (VEOG) eye movements were recorded. An online bandpass filter was set with limits at 0.1 and 30 Hz. Automatic artefact rejection was applied offline to exclude all epochs with voltage exceeding ±80 µv. Offline the recordings were re-referenced to the average of all electrodes (except for HEOG and VEOG) and a spline fit was used to convert to a sampling rate of 250 Hz.

### Computation of the intraclass correlation (ICC)

Computation of the ICC is described in detail by Bishop *et al.* (2007). In brief, the ICC is similar to the more familiar Pearson correlation co-efficient, but is sensitive to amplitude as well as shape. The one-way random effects ICC (Shrout & Fleiss, 1979) was computed between datapoints between two waveforms: a *normative*waveform, which was the grand average for the control group, and a *comparison* waveform, which is the waveform of an individual child. To ensure total independence of normative and comparison waveforms, the comparison waveform was never included in the average used for the normative waveform: i.e. when computing the ICC for a typically developing child, the normative grand average was based on all the other control children. Note, however, that normative waveforms formed by dropping one subject from a sample of 47 were virtually identical.

## Results

### Comparison of ICCs for SLI and typically developing children

**Figure 1:**
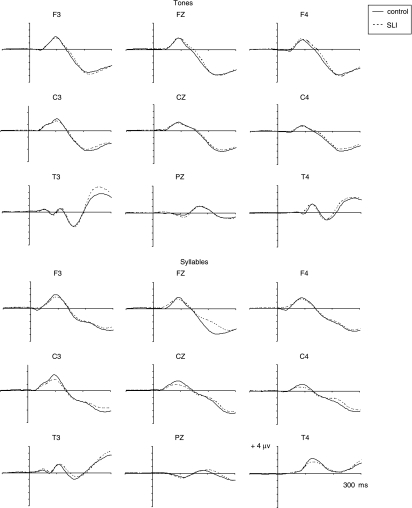
Grand average auditory ERPs for tones (upper panel) and syllables (lower panel) from typically developing control (continuous) and SLI (dashed) groups at nine electrode sites.

Children aged from 5 to 10 years were treated as a single group because there was no evidence of any developmental change in this age range (Bishop *et al.*, 2007). Figure 1 shows the grand average auditory ERP for SLI and control groups at nine electrodes: F3, FZ, F4, C3, CZ, C4, PZ, T3 and T4, for both tone and speech stimuli. The overall waveform shape and peak latencies appear similar for the two groups, with only minor differences evident in amplitude at some electrodes. ICCs with control grand means were computed for all individuals across the time scale 100 to 228 ms (33 data points) at these electrodes. This interval was selected so that we could directly compare results with those of Bishop and McArthur (2005), who selected this interval because it encompassed N1 and P2 for those individuals who had these peaks.

**Figure 2:**
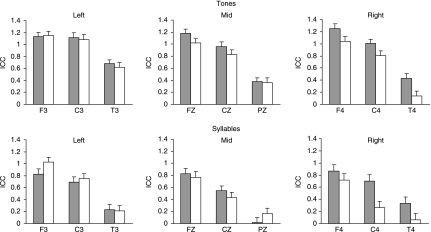
Mean Fisher-transformed ICC values for SLI (white bars) and control children (grey bars) at nine electrode sites for tone and syllable stimuli.

ICCs were transformed to Fisher *z* to normalize the data, and then compared in a mixed ANOVA with two repeated measures (electrode: F3, Fz, F4, C3, Cz, C4, Pz, T3 and T4, and material: tone, speech) and one between-subjects variable, group (SLI vs. control). Mean Fisher-transformed ICCs at each electrode are shown in Figure 2.

Mauchly's test indicated that the assumption of sphericity was violated for the main effects of electrode (χ^2^= 288.7, d.f. = 35, *p* < .001). Degrees of freedom were therefore corrected using Greenhouse-Geisser estimates of sphericity (ɛ= .59).

The main effect of group was non significant; *F*(1, 87) = 2.61, *p*= .109. There were significant effects of stimulus, indicating that ICCs were generally higher for tone than for syllable stimuli: *F*(1, 87) = 67.3, *p* < .001. There was a significant effect of electrode: *F*(4.71, 409.6) = 53.4, *p* < .001. In interpreting these main effects, note that the dependent variable is a measure of agreement between an individual's waveforms and those of the grand average control group, rather than absolute amplitude of the ERP. A high ICC indicates greater uniformity in the waveforms of the sample, whereas a lower mean ICC indicates that the auditory ERP is more variable from child to child.

The interaction between stimulus and group was not significant (*F* ratio < 1). However, there was a significant interaction between electrode and group: *F*(4.7, 409.6) = 2.93, *p*= .015. This was further investigated using post-hoc one-tailed *t*-tests (87 d.f.) to compare the mean ICC (average of both conditions) of groups for each electrode, after first establishing that Levene's test of equality of variances was not violated at any electrode. The SLI group obtained significantly lower ICCs for tone stimuli at electrodes F4 (*t*= 1.87, *p*= .032), C4 (*t*= 2.81, *p*= .003) and T4 (*t*= 2.86, *p*= .003).

### Analysis of mean amplitude over 100–228 ms

To check whether the same pattern of results would be obtained if one considered mean amplitudes rather than ICCs, an ANOVA was conducted on the factors group, stimulus, and electrode with mean amplitude as dependent variable. The main effect of group and all interactions with group fell well short of significance (*F* < 1). It is clear that the group differences observed with the ICC do not simply reflect group differences in mean amplitude in the N1 interval.

### Characteristics of children with atypical ERPs

Although there were significant differences in ICC values, there was nevertheless substantial overlap between groups, suggesting that only a subset of children with SLI have atypical ERPs. To explore whether this subgroup had specific characteristics, children were subdivided according to their ICC values at electrode C4 with speech stimuli, this being the electrode and condition that gave the largest group difference. Eight of 47 (17%) control children obtained an ICC of zero or less; this value was taken therefore as defining the lower bound of normal limits (approx. 1 SD below the mean). Fourteen of 42 (33%) children with SLI had an ICC this low, and nine of these scored below the lowest ICC of any control (less than −0.3). This ‘low-ICC’ subgroup was compared with the remainder of the SLI group (‘average-ICC’ subgroup) and found not to differ significantly on age, gender, handedness or nonverbal IQ, or on number of epochs included in the ERP averages (see Table 2). Uwer *et al.* (2002) had found higher error rates in children with SLI vs. controls in tests of discriminating tone frequency, tone duration, and syllable identity, but there was no hint of higher error rates in the ‘low ICC’ subgroup on these behavioural measures. However, the subgroups did differ in their SLI classification, with the excess of low-ICC cases being confined to those with receptive SLI. Ten of 21 (48%) children with receptive SLI had a low ICC, compared with four of 21 (19%) of the expressive SLI children.

**Table 2:** Characteristics of SLI sample, subdivided according to whether ICC to syllables at C4 is above 0 (average) or below this level (low)

	Average ICC *N*= 28		Low ICC *N*= 14				
					
Variable	Mean	SD	Mean	SD	*t*	d.f.*	*p*
*N* epochs tones	753.0	116.3	781.5	86.4	−1.18	40	.243
*N* epochs syllables	759.3	124.4	772.4	83.7	.71	34	.480
Age (months)	95.2	13.2	100.4	13.3	−.35	40	.726
Nonverbal IQ	101.1	10.7	99.8	11.4	−.81	40	.423
Auditory discrimination errors							
Frequency	10.7	8.6	6.2	6.1	1.65	36	.108
Duration	17.8	9.3	15.4	9.2	.38	40	.707
Speech	9.7	12.0	6.3	6.0	.93	32	.360
	%		%		χ^2^		*p*
% male	21.4		21.4		0		1.00
% non-right handed	22.2		35.7		.86	1	.36
% receptive SLI	39.3		71.4		3.86	1	.05

* Some missing data for auditory discrimination tasks: Frequency: N1 = 25, N2 = 13; Duration: N1 = 25, N2 = 11; Speech: N1 = 22, N2 = 12.

**Figure 3:**
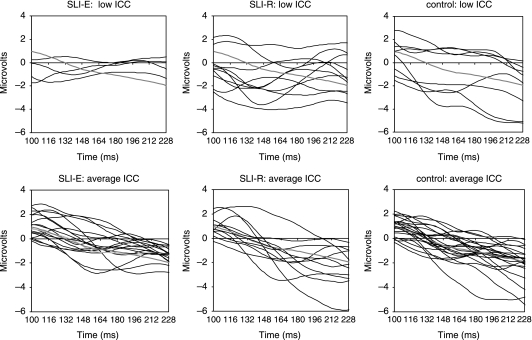
Individual waveforms at electrode C4 to syllable stimuli in the time window 100 to 228 ms, for typically developing and SLI subgroups, subdivided according to ICC for speech at C4. SLI-E is expressive SLI; SLI-R is receptive SLI. The grey line shows the normative grand mean.

The significant group differences in ICC appear at first sight to be at odds with the impression from the grand average waveforms shown in Figure 1, which suggest that any differences between SLI and control children are slight. However, further investigation indicated that the grand averages are misleading, because they mask quite substantial variability within the group. This was explored further by subdividing children according to their SLI classification and their ICC for syllable stimuli at electrode C4. Figure 3 shows indi-vidual waveforms plotted against the normative grand mean for speech stimuli in each subgroup. On visual inspection, it can be seen that for most of those with an average ICC there is a downward slope in the waveform between 100 and 228 ms. This also appears to be the case for control children with low ICCs, where the low ICC seems to reflect large differences in amplitude from the normative grand mean, rather than differences in shape. However, for SLI children with low ICCs, individual waveforms over this time range do not show such a clear negative slope. Whereas virtually all children with average ICCs have positive amplitudes at the start of the interval, most of the SLI cases with low ICCs have negative amplitudes at this time point.

### Topographic maps

**Figure 4:**
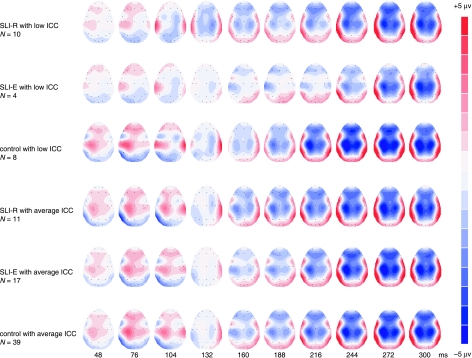
2-D cartoons showing brain responses to syllables in time window 48 to 328 ms for typically developing and SLI subgroups, subdivided according to ICC for speech at C4.

To gain further insight into the nature of the group differences, 2-D topographic maps were created for the grand average ERP to syllables over an interval encompassing P1-N2 (48 to 328 ms) for typically developing and SLI children, subdivided according to ICC for syllables at electrode C4. These maps must be treated cautiously, as distributions of activation can look very different depending on the amplitude and time ranges that are used, and amplitudes at surface electrodes are not a good indication of the source of underlying activity. Furthermore, the grand average may not be representative of individuals that contribute to it. Nevertheless, they may elucidate topographic alterations in activation that are hard to see from the raw waveforms. As can be seen in Figure 4, brainmaps for the SLI group with average ICC are closely similar to the typically developing group. Confirming the picture from Figure 3, the grand average for the SLI subgroups with low ICC are markedly different, with a pronounced early negativity starting around 100 ms at right central sites. In the receptive SLI subgroup this spreads to encompass a wide fronto-central region in the next time slice, at a point when other groups are only beginning to develop signs of negativity in these regions. Another unusual feature in the low-ICC receptive SLI subgroup is bilateral positivity in temporal regions around 132 ms, at a time when other groups show positivity lateralized to right temporal electrodes. Analogous brainmaps were also derived for ERPs to tone stimuli for the same subgroups (see Supplementary material on http://www.psy.ox.ac.uk/oscci/dbhtml/Excelequals;percnt;20files/supplementaryequals;percnt;20plots.xls), and again showed anomalous right central negativity around 100 ms in the low-ICC receptive SLI subgroup.

## Discussion

Using a sample of children aged from 5 to 10 years, we compared each individual's waveform in the range 100 to 228 ms with that of a normative grand average. The analytic method was based on Bishop and McArthur (2005): rather than attempting to measure the amplitude and latency of peaks, we used the ICC to provide a global measure of similarity between the waveform of an individual child and the grand average of a typically developing group of the same age. We found that the waveforms of children with SLI showed abnormalities (i.e. relatively low ICCs) for frontal, central and temporal electrodes on the right side of the head, with a trend for smaller differences at the midline. No differences were seen for electrodes on the left side of the head. The finding of significant group differences in the time window 100–228 ms is consistent with results obtained with older children by Bishop and McArthur (2005), although they found significant differences at electrode Fz, whereas we saw only a nonsignificant trend at this site.

Despite this group difference, a substantial proportion of children with SLI had age-appropriate waveforms in this time interval. These findings are similar to those of Tonnquist-Uhlén *et al.* (1996), who noted that many children with SLI had a normal N1, but nevertheless the proportion with atypical findings was higher than in controls.

### Which children have atypical waveforms?

This study confirms suggestions that children with SLI are heterogeneous, with some showing normal auditory ERPs and others differing from controls (Neville *et al.*, 1993; Tonnquist-Uhlén *et al.*, 1996). If atypical waveforms characterize only a subset of those with SLI, then we will need large samples in order to have sufficient power to detect group differences, yet many studies in this area include 12 or fewer. One way forward is to identify the characteristics of those children with atypical waveforms, in order to reduce heterogeneity in future studies.

The only difference that we found between children with typical and atypical waveforms was in terms of subtype of SLI: those with receptive SLI were more likely than controls to have atypical waveforms, whereas this was not the case for children with purely expressive problems.

One might expect that the link between atypical auditory ERPs and receptive SLI might be an indicator of abnormal function of auditory cortex leading to poor speech discrimination; however, like McArthur and Bishop (2005), but in contrast to Neville *et al.* (1993), we did not find that abnormal performance on auditory tasks characterized those children who had atypical waveforms. However, it must be noted that the auditory assessments used with this sample were fairly limited. More studies are needed to clarify the functional significance for auditory and language processing of having an atypical auditory ERP. As argued below, it is possible that the atypical ERPs reflect unusual patterns of underlying brain morphology. If so, these may be correlated with SLI without having any significance for auditory discrimination skills.

### What does an atypical waveform signify?

The ICC analysis, coupled with overall scrutiny of the brain maps, suggests that there is atypical lateralization of brain responses to sounds in some children with SLI. The laterality effect (lower ICCs in SLI on the right but not the left fronto-central regions) is consistent with several other studies that have reported anomalous cerebral lateralization in some cases of SLI using measures of brain structure (Gauger, Lombardino & Leonard, 1997; De Fossé, Hodge, Makris, Kennedy, Caviness, McGrath, Steele, Ziegler, Herbert, Frazier, Tager-Flusberg & Harris, 2004; Herbert, Ziegler, Deutsch, O’Brien, Kennedy, Filipek, Bakardjiev, Hodgson, Takeoka, Makris & Caviness, 2005) or function (Mason & Mellor, 1984; Dawson, Finley, Phillips & Lewy, 1989; Chiron, Pinton, Masure, Duvelleroy-Hommet, Leon & Billard, 1999; Shafer, Schwartz, Morr, Kessler & Kurtzberg, 2000). Atypical lateralization of ERPs has also been reported in infants who have a family risk for SLI (Benasich, Choudhury, Friedman, Realpe-Bonilla, Chojnowska & Gou, 2006). Also, unusually large, positive, right-hemisphere responses to /ga/ were found in infants who subsequently turned out to have poor receptive language (Guttorm, Leppänen, Poikkeus, Eklund, Lyytinen & Lyytinen, 2005).

There are three broad classes of explanation for such an effect. The first, which we may term the ‘atypical brain’ hypothesis, maintains that the brain is not optimally wired up for language learning. According to this view, brain organization in some children with SLI is qualitatively different from that in typical development, presumably because of genetic influences on prenatal brain development. An alternative ‘epigenetic’ hypothesis maintains that brain differences between children with SLI and controls are not a cause but a *consequence* of the language disorder: if verbal signals are not meaningful to the child, then these may be tuned out, and brain networks for processing language may show aberrant development. Circumstantial evidence against this explanation comes from our findings that (a) only a subset of cases with receptive SLI have atypical ERPs and (b) abnormal auditory ERPs are seen in response to nonverbal as well as verbal sounds. A final ‘maturational lag’ hypothesis maintains that electrophysiological differences in auditory responses of children with SLI vs. controls are indicators of neurodevelopmental immaturity, rather than any stable differences in underlying brain structure. For instance, we know that the cerebral commissures continue to myelinate throughout childhood (Giedd, Rumsey, Castellanos, Rajapakse, Kaysen, Vaituzis, Vauss, Hamburger & Rapoport, 1996). It is possible that the differences in lateralization between receptive SLI and typically developing children reflect some delay in maturation of interhemispheric transmission: this kind of explanation has been proposed for learning disabilities, though it is highly speculative (Njiokiktjien, de Sonneville & Vaal, 1994; Von Plessen, Lundervold, Duta, Heiervang, Klauschen, Smievoll, Ersland & Hugdahl, 2002). Bishop and McArthur (2005) argued for a maturational lag account on the basis that the auditory ERPs of adolescents with SLI were more similar to those of younger typically developing children than to controls of their own age. We could not replicate this finding here, because all the children with SLI in the current study were aged between 5 and 10 years, an interval over which the ICC did not reveal any changes with age in the auditory ERP (see Bishop *et al.*, 2007). Additional studies comparing children with SLI with younger typically developing children in the same paradigm would be necessary to establish whether the pattern seen in our low-ICC subgroups is immature, but this seems unlikely. In a study that was similar to ours in terms of stimuli (150 ms duration 1000 Hz tones) and presentation rate (SOA of 900 ms), Morr, Shafer, Kreuzer and Kurtzberg (2002) examined auditory ERPs in children aged from 2 to 47 months. In all groups, the dominant observation around 100 ms was a *positivity* which increased in magnitude over the observed age range. Thus the fronto-central negativity around 100 ms in children with receptive SLI appears to be abnormal rather than developmentally delayed. It is possible that N100 in these children is unmasked because other components are missing (cf. Ponton *et al.*, 2000). There would be considerable interest in repeating our study using longer or shorter SOAs, to see whether group differences emerge more strikingly at slow presentation rates when N1 can be reliably elicited.

Additional studies with children with SLI aged 12 years and over would also be of interest, as we know that the auditory ERP changes radically around adolescence (see Bishop *et al.*, 2007). A maturational account suggests that children with SLI might show a shift to a more mature pattern of ERP at a later age than usual.

### Implications for ERP studies of developmental disorders

It is noteworthy that the differences uncovered here would not have been detected had we relied on measures of peak amplitude and latency because so many children did not have the N1 and P2 peaks. As we have shown, mean amplitude measures also failed to detect the abnormalities found here, because they are insensitive to waveform shape. It is possible to get information on waveform shape without identifying peaks by dividing an interval (such as the 100–228 ms one used here) into a number of consecutive windows and computing mean amplitude in each of these. However, this method has several disadvantages: the size of window is arbitrary, yet can have a dramatic effect on results, and the high interdependence of consecutive windows creates problems for statistical analysis. Perhaps most importantly, a multiple-window approach does not provide a ready way of identifying those children who have atypical waveforms, and will only reveal significant group differences if atypical waveforms are all atypical in the same way. The ICC is especially useful when there is heterogeneity in the ERPs of individuals, as it can identify abnormality, even when the specific type of abnormality differs from case to case. ICC is a global measure that does not specify the nature of differences between waveforms; however, we suggest that this study proves its usefulness in highlighting cases of atypical auditory ERPs, which may then be explored by more detailed consideration of waveform shape and topography.

Our analysis highlights a drawback of grand averages which is that they obscure individual differences. Using the ICC we found subtle differences in the late auditory ERP in just a subset of cases. When individual waveforms vary in the timing of peaks and troughs and in their amplitudes, group differences will be masked when a grand average is computed. The ICC provides a quantitative estimate of the abnormality of a waveform that has promise as a means of analysing individual differences.
